# Expression of SIRPα-Fc by oncolytic virus enhances antitumor efficacy through tumor microenvironment reprogramming

**DOI:** 10.3389/fimmu.2025.1513555

**Published:** 2025-02-25

**Authors:** Qingzhe Yang, Yongheng Shu, Yanwei Chen, Zhongbing Qi, Shichuan Hu, Yao Zhang, Yu Qin, Xianglin Xu, Jianchuan Hu, Anliang Huang, Ping Cheng

**Affiliations:** ^1^ Department of Biotherapy, Cancer Center and State Key Laboratory of Biotherapy, West China Hospital, Sichuan University, Chengdu, China; ^2^ Department of Pathology, Chengdu Fifth People’s Hospital, Chengdu, China

**Keywords:** tumor-associated macrophages, oncolytic adenovirus, SIRPα, tumor immunotherapy, tumor microenvironment

## Abstract

Oncolytic viruses (OVs) selectively replicate within tumors, directly killing cancer cells and promoting a systemic immune response by releasing tumor antigens. These features make OVs a promising approach in tumor immunotherapy, offering targeted treatment with fewer side effects. Despite these advantages, OVs are primarily administered via intratumoral injection, limiting their effectiveness for advanced, systemic cancers. Among OVs, oncolytic adenoviruses (oAdVs) are the most widely studied due to their well-understood gene regulation, safety, and stability. In this study, a modified oAdV vector, pDC316-oAd-SA, was engineered to express the SIRPα-mIgG1Fc gene, designed to remodel tumor-associated macrophages (TAMs) and enhance anti-tumor immunity. This vector, along with a control virus (Ad-ON), was evaluated both *in vitro* and *in vivo*. The modified oAd-SA significantly improved macrophage phagocytosis and showed superior tumor regression in murine models. Additionally, while both oAdVs increased T cell infiltration in the tumor microenvironment, oAd-SA specifically enhanced T cell immune function. The study also revealed that oAdVs modulate TAMs differently across tumor types, with oAd-SA therapy particularly increasing TAM phagocytosis and promoting an anti-tumor response.

## 1 Introduction

Oncolytic viruses (OVs) have emerged as promising therapeutic agents for cancer, showing selective replication in tumor cells, leading to their destruction. This process also triggers immune responses that enhance the body’s defense against cancer. Extensive research has highlighted the potential of OVs in preclinical and clinical studies to transform cancer therapy (1–6).Adenoviruses, among the most potent OVs, are well-known for their genetic stability and non-integration into the host genome, eliminating the risk of genotoxicity. Their high gene delivery efficiency and ability to recruit immune cells while inhibiting regulatory T cells in the tumor microenvironment make them ideal candidates for cancer treatment (7–11). However, clinical trials show that adenovirus monotherapy alone may not eliminate tumors (12).To overcome this, researchers are exploring combinations with other cancer treatments or adding therapeutic transgenes to enhance the antitumor effects of adenoviruses (13–17).

Tumors have developed complex strategies to evade the immune system, such as creating an immunosuppressive microenvironment and avoiding immune detection. One key mechanism of immune evasion involves the signal-regulatory protein α (SIRPα), found on myeloid cells like macrophages and dendritic cells. Its cytoplasmic tail contains immunoreceptor tyrosine-based inhibition motifs (ITIMs), which help modulate immune suppression. Tumor cells overexpress CD47, a transmembrane protein, to bind SIRPα and send a ‘do not eat me’ signal, preventing phagocytosis and allowing tumors to avoid immune destruction. This makes the CD47-SIRPα interaction a promising target for cancer immunotherapy. Blocking this interaction has shown potential in restoring macrophage phagocytic activity and killing tumor cells (18–23).

Several antibodies targeting the CD47-SIRPα pathway are currently under investigation in clinical trials. The humanized CD47 antibody Hu5F9-G4 (Magrolimab) has demonstrated enhanced phagocytosis of tumor cells *in vitro* and therapeutic effectiveness *in vivo*, particularly in acute myeloid leukemia (AML) and myelodysplastic syndromes (MDS) (24). ADU-1805, a humanized monoclonal IgG2 anti-SIRPα antibody, promotes macrophage phagocytosis and neutrophil trogocytosis, and is being evaluated for solid tumors (25). IMM0306, a fusion protein that combines CD20 mAb with the CD47-binding domain of SIRPα, activates both macrophages and NK cells, currently undergoing trials in a variety of cancer types (26). KWAR23, an anti-SIRPα antibody, significantly enhances the antitumor activity of neutrophils and macrophages when paired with tumor-opsonizing antibodies and is in clinical testing (27). Ongoing phase I trials are exploring bispecific antibodies and combination therapies targeting the SIRPα-CD47 interaction to bolster anti-tumor immunity (28). Adverse effects, particularly hemolysis, are primarily associated with CD47 antibodies rather than anti-SIRPα antibodies (29). Some studies indicate that recombinant SIRPα or anti-CD47 antibodies may lead to anemia but do not specifically attribute intravascular hemolysis to SIRPα antibodies (30). Research on sickle cell anemia highlights CD47 blockade without detailing specific adverse effects of SIRPα antibodies (31). Additional findings suggest that anti-CD47 antibodies with effector function could result in hemolysis, while anti-SIRPα antibodies show minimal binding to erythrocytes, thereby reducing potential toxicity (32, 33).

Oncolytic viruses (OVs) represent a compelling class of agents for the treatment of various malignancies, selectively replicating in and lysing tumor cells (34, 35) while eliciting both innate and adaptive immune responses (36, 37). A diverse array of OVs has been examined in preclinical and clinical studies (38, 39), with adenoviruses emerging as some of the most extensively utilized due to their advantageous properties (40, 41). As double-stranded DNA viruses, adenoviruses are genetically stable and do not integrate into the host genome, thereby minimizing genotoxicity. Their remarkable gene-delivery efficiency further amplifies their therapeutic potential (42). Additionally, adenoviruses can reshape the tumor microenvironment by recruiting CD45+ leukocytes and CD8+ lymphocytes while inhibiting FoxP3+ lymphocyte infiltration (43, 44). However, past experiences suggest that monotherapies utilizing oncolytic adenoviruses may not completely eradicate tumors (45). To enhance their efficacy, these viruses are frequently combined with other cancer treatments or engineered with therapeutic transgenes (46–49).

In this study, we constructed an oncolytic adenovirus designed to express an engineered SIRPα variant with an IgG1 Fc protein and investigated its therapeutic efficacy against CD47^+^ murine tumor cell lines both *in vitro* and *in vivo*.

## 2 Materials and methods

### 2.1 Cell lines

Human embryonic kidney 293 (HEK293) and 293A, murine colon carcinoma cell line MC38, murine melanoma cell line B16-F10, murine mammary carcinoma cell line 4T1 and murine lymphoma cell line were obtained from American Type Culture Collection (ATCC). HEK293, 293A and A20 cell lines were grown in Dulbecco’s modified Eagle’s medium (DMEM) supplemented with 10% fetal bovine (FBS) and maintained at 37°C in 5% CO_2_, while B16-F10, MC38 and 4T1 cell lines were cultured in Roswell Park Memorial Institute 1640 (RPMI 1640) supplemented with 10% FBS in the same condition.

### 2.2 Virus preparation

In a meticulously orchestrated process, a fragment of the engineered SIRPα variant fused with the Fc gene (hereafter referred to as SA) was synthesized by the adept hands of GENEWIZ Biotech, located in Suzhou, China. The plasmid pDC316-hTERT-E1AE1B (pDC316-oAd), a critical component of our experimental framework, underwent precise enzymatic digestion with SalI. This enzymatic action was followed by a strategic ligation of the digested product with the SA fragment, culminating in the creation of the recombinant plasmid pDC316-oAd-SA. The pDC316-oAd-SA plasmid, now a beacon of our genetic engineering prowess, was then allied with an adenoviral backbone plasmid, pBHBlox(delta)E1-3cre, and the union was transfected into the robust HEK293A cell line. This transfection set the stage for the viral drama to unfold, with the cells diligently working to produce viral progeny. After a period of approximately 7 to 14 days post transfection, the diligent observation of the cellular culture yielded the sighting of multiple viral plaques within the HEK293A cells—a testament to the successful replication of our engineered virus. These plaques were harvested with care and underwent rigorous verification through the gold-standard polymerase chain reaction (PCR) analysis to confirm their identity. The recombinant adenovirus, now confirmed and proudly named oAd-SA, was allowed to propagate in the hospitable environment of HEK293 cells. Subsequently, it was subjected to a purification ritual involving ultracentrifugation through discontinuous cesium chloride (CsCl) gradients—a process that separated the viral particles from the cellular debris and other impurities with high fidelity. Consistent with our commitment to methodological rigor, other viruses utilized in our study were packaged and treated with an equivalent level of precision and care. After determining the viral titer, which is a measure of virus concentration, the viruses were meticulously portioned and conserved in the icy embrace of -80°C, ensuring their potency and readiness for future applications.

### 2.3 *In vitro* viral infection and cell cytotoxicity assay

4T1 and MC38 cells were plated in 24-well plates and infected with oAd-SA compared to oAd-ON at an MOI of 0, 1, 5,10,20 and 50. The cells were stained with crystal violet staining solution (Sigma) for 5 min after the different time of the infection.

4T1 and MC38 cells were plated in 96-well plates and infected with oAd-SA compared to oAd-ON at an MOI of 0, 1, 5,10,20 and 50. Cytotoxicity was evaluated by using CCK8 after the different time of the infection.

### 2.4 Validation of oAd-SA virus

The purified virus was used to infect 4T1, MC38, CT26, and B16-F10 cells at an MOI of 20. After 2 days, cell pellets were collected and washed twice with PBS. The pellets were then divided into two portions. One portion was lysed with TRIzol for 30 minutes to extract RNA from the cell sediment. The other portion was lysed with RIPA buffer containing protease inhibitors for 30 minutes to extract proteins from the cell sediment. This process will facilitate the subsequent detection of the target gene SIRPα-Fc expression.

### 2.5 Binding assay

Cells (A20, 4T1, and MC38) were individually collected into flow cytometry tubes, washed with 1× PBS, resuspended in 100 μl of 1× PBS, and incubated with CD47 flow cytometry antibody. After antibody removal by washing, CD47 expression on tumor cells was measured using flow cytometry. Concurrently, cells were seeded at 1×10^5^ cells/well in a 24-well plate, treated with oAd-ON or oAd-SA viruses at MOI 50, and incubated for 2 days prior to cell collection. Meanwhile, CD47 expression on tumor cells was measured using WB.

### 2.6 Isolation and culture of bone marrow-derived macrophages

Euthanize a C57 or BLAB/c mouse using cervical dislocation. Remove the hind legs with sterile scissors and tweezers, ensuring to remove attached muscles. Wash the bones twice with 5 ml of ice-cold sterile 1x PBS. Use a 1 ml syringe to flush cells from the femur and tibia to obtain a cell suspension. Filter the cell suspension through a 70 μM filter and centrifuge at 450 x g for 10 minutes to collect the cell pellet. Resuspend the cell pellet in 5 ml of red blood cell lysis buffer and incubate for 2 minutes until the suspension loses its red color. Add 10 ml of complete DMEM medium to stop the lysis, then centrifuge at 450 x g for 10 minutes. Discard the supernatant. Resuspend the cell pellet in 20 ml of pre-warmed 10% FBS 1640 medium, and transfer to a 10 cm culture dish. Incubate in a 5% CO_2_ incubator. Change the medium after 3 days of culture and continue to culture for an additional 2 days. Perform flow cytometry antibody detection to verify cell purity and phenotype before use.

### 2.7 *In vitro* phagocytosis assay and co-culture experiments

Bone marrow derived macrophage (BMDM) was isolated from femur and tibia of Balb/c mice and confirmed by flow cytometry. 24h before treatment, 1x10^5^ A20, 4T1 and MC38 cells were seeded in 12-well plate, three wells for each cell. The cells were infected by Ad-ON or Ad-SA with multiplicity of infection of 50 (MOI=50). Wells with medium only was taken as control. 2 days later, above tumor cells were collected, stained with PKH26, reseeded in 24-well plate. Then, 5x10^4^ BMDM stained with Crystal Field Stabilization Energies (CFSE) was added into each well. This was followed by incubation for 3.5 h in the incubator. Finally, the cells mixture was collected, and phagocytosis was evaluated via flow cytometry and confocal microscopy. After co-culturing tumor cells with macrophages, F4/80 was used to label the macrophages to assess the effect of oAd-SA on macrophage polarization, with CD86 serving as a marker for M1-type macrophages.

### 2.8 Animal experiment

The 6-8 weeks old female Balb/c and C57BL/6 mice were purchased from Huafukang Bioscience (Beijing, China). All the animal experiments were approved by Institutional Animal Care and Use Committee Sichuan University. Briefly, 1×10^6^ 4T1 or A20 cells was implanted subcutaneously on the right flank of Balb/c mice and 1×10^6^ MC38 cells was inoculated at the same position of C57BL/6 mice to establish xenografts, respectively. The mice were randomly divided into different groups based on experimental arrangement before receiving any treatment. In brief, mice from Ad-SA group were intratumorally injected 5×10^8^ PFU Ad-SA in 50ul PBS every three days when tumor reached 50-100mm^3^ on average, 3 doses were administrated in total. Mice from Ctrl and Ad-ON group received same volume of PBS or same dosage of Ad-ON, respectively. During the experiment, tumor size was measured by a caliper and determined according to formula:


$$Tumor\;Size=L*W^{2}*0.5236$$

Where L and W represent the length and width of the tumor, respectively.

### 2.9 Flow cytometry

Animals were euthanized 4 days after the administration of adenovirus. Tumor and spleen were harvested. For tumor cells analysis, about 100mg of tumor was minced into small pieces and submerged in RPMI-1640 (Gibco) with 0.1% (w/v) IV collagenase and 1% FBS at 37°C for one and a half hours with agitation, filtered with 70-um sieve, washed and resuspended in PBS. Then stained with Fixable Viability Stain 620 to exclude dead cells. The cells were blocked by Fc-block (BD biosciences) and stained with antibodies. For nuclear factors detection like FoxP3, fixation and permeabilization kit (eBioscience) was used. For spleen cells analysis, the spleen was grinded through 70-um sieve, then cells were extracted with lymphocyte separation medium (BD bioscience) according to manufacture instructions and stained with antibodies. For intracellular factors analysis such as IFNγ, fixation and permeabilization solution (BD bioscience) was utilized. Cells were analyzed on a NovoCyte flow cytometer. Antibodies to CD3 (145-2C11), CD4 (RM4-5), CD8 (53-6.7), CD45 (30-F11), CD11b (M1/70), F4/80 (BM8), CD206, Gr1 (RB6-8C5), CD25 (3C7), FoxP3 (MF-14), CD86 (GL-1), IFN-γ and TNF-α were acquired from Biolegend.

### 2.10 *In vivo* phagocytosis assay

Frozen sections of tumor tissue were obtained, with nuclei stained using DAPI, CK19^+^ cells labeled in green fluorescence, and F4/80^+^ cells in red fluorescence. Similar to the *in vitro* phagocytosis assay, yellow fluorescence (resulting from the overlap of red and green) indicates active phagocytosis. Immunofluorescence was utilized to observe *in vivo* phagocytosis.

### 2.11 Statistical analysis

Data were analyzed using GraphPad Prism. Statistical significance was determined using unpaired t-tests. Animal survival was illustrated using Kaplan-Meier survival curves and analyzed using the log-rank test. Immunohistochemistry and immunofluorescence images were quantified using Image Pro Plus 6.0. Data are presented as mean ± SEM. A p-value of <0.05 was considered statistically significant. In figures, symbols denoting significance are: *p < 0.05, **p < 0.01, ***p < 0.001, and ns (no statistical significance).

## 3 Results

### 3.1 Evaluation of SIRPα-Fc expression and adenoviral sensitivity in tumor cell lines

**Figure 1:**
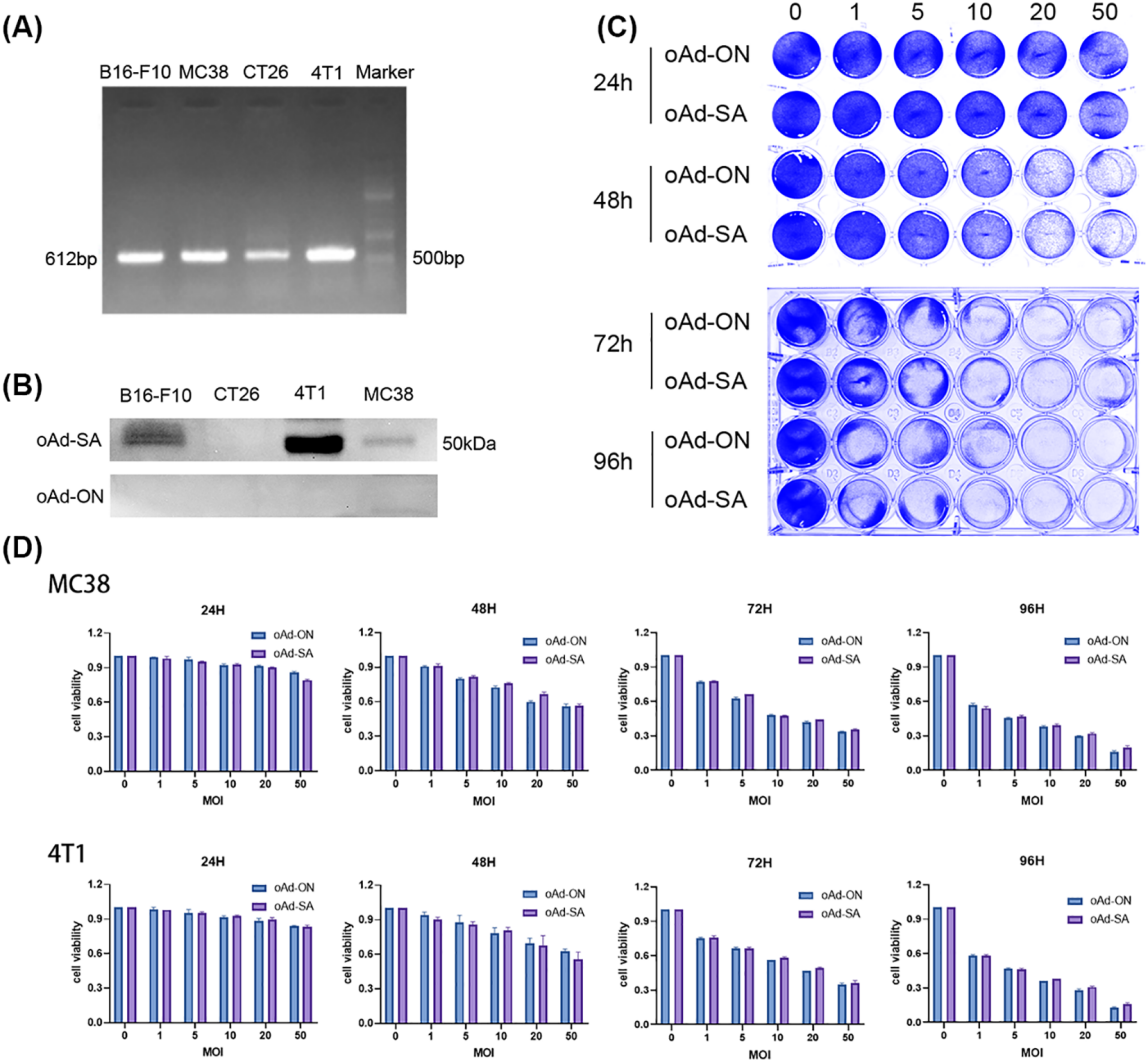
Analysis of SA gene expression and adenoviral sensitivity in tumor cell lines. **(A)** RT-PCR analysis showing a prominent band at 612 bp for the SA gene. CT26 cells exhibit a weaker band compared to 4T1, MC38, and B16-F10 cells. **(B)** Western blot analysis of cell pellets (4T1, MC38, B16-F10, and CT26) displaying SIRPα-mIgGFc protein at approximately 50 kDa. **(C)** The following schematic diagram illustrates the representative crystal violet staining. **(D)** Dose-dependent effects of infection at varying multiplicities of infection (MOI=0, 1, 5, 10, 20, 25) on cell density and viability of 4T1 and MC38 cells.

RT-PCR analysis revealed a prominent band at 612 bp for the SA gene, as shown in **Figure 1A**. Interestingly, the reverse transcription amplification from CT26 cells exhibited a weaker band compared to the others. **Figure 1B** displays the results of a Western blot analysis on the cell pellets of 4T1, MC38, B16-F10, and CT26, indicating the presence of SIRPα-mIgGFc protein at around 50 kDa. The intensity of the protein bands was strongest in 4T1 cells, followed by B16-F10 and MC38, while CT26 showed no significant protein band. This weaker expression in CT26 may be attributed to its lower sensitivity to adenovirus, resulting in fewer viral particles entering the cells and consequently a diminished expression of the target gene. Additionally, nucleic acid detection methods generally exhibit higher sensitivity compared to protein detection.

In an *in vitro* virus infection assay, we found that 4T1 and MC38 cells demonstrated strong sensitivity to adenovirus, leading us to construct tumor models for subsequent *in vivo* experiments. The oAd-SA demonstrated a significant inhibitory effect on tumor cell growth, with infection of tumor cells at varying multiplicities of infection (MOI = 0, 1, 5, 10, 20, 25) resulting in a dose-dependent decrease in both cell density and viability, as illustrated in **Figures 1C, D**.

### 3.2 Infection of oAd-SA strengthens the phagocytosis of macrophage against tumor cells *in vitro*

**Figure 2:**
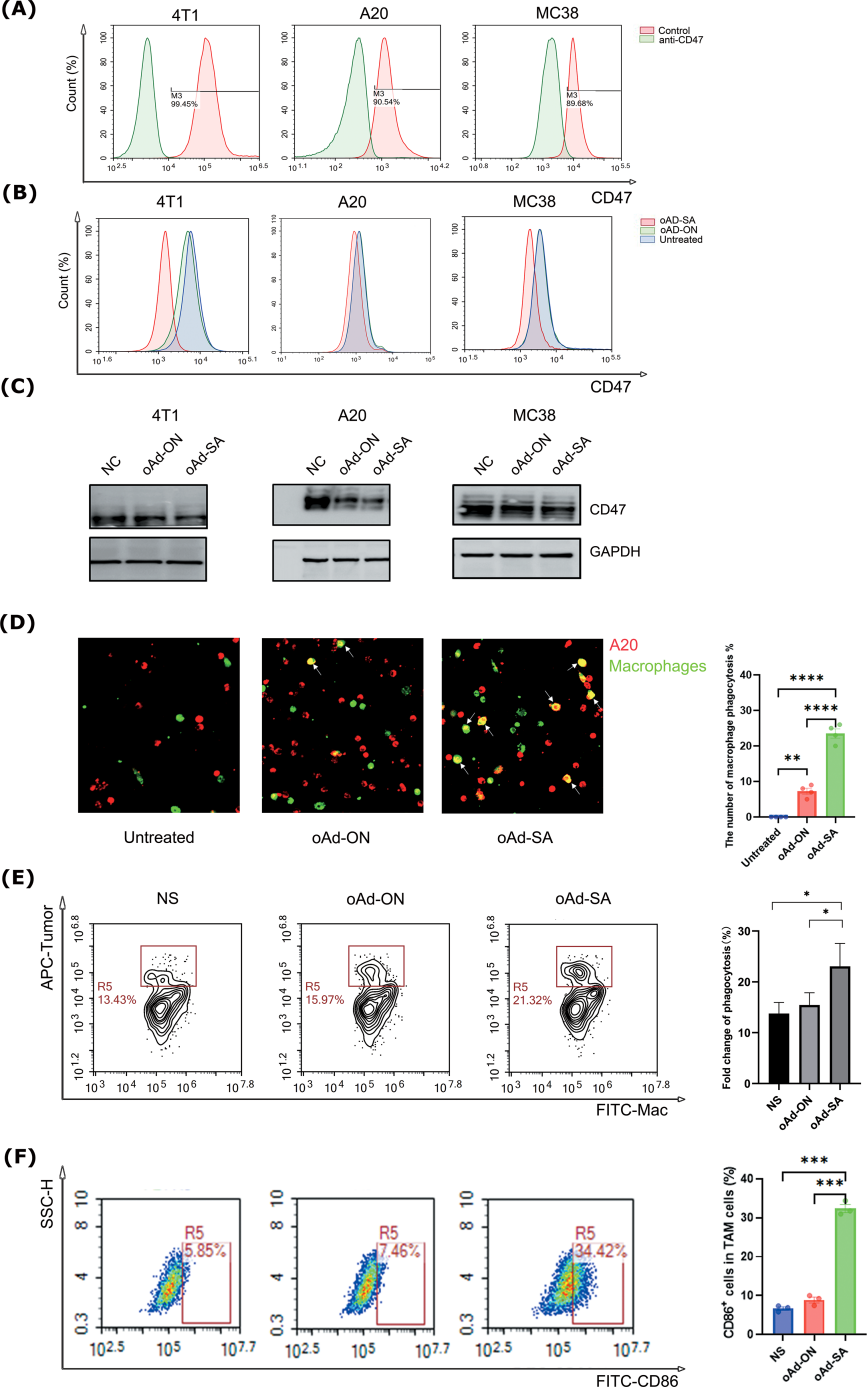
Infection of oAd-SA decreases CD47 expression on tumor cells and strengthens the phagocytosis of macrophages against tumor cells *in vitro*. **(A)** Flow cytometric analysis of CD47 expression levels in various tumor cell lines. **(B)** Flow cytometric analysis of CD47 expression in untreated tumor cells and after oAd-ON and Ad-SA infection. **(C)** WB detected the CD47 expression in different tumor cells after oAd-ON and Ad-SA infection **(D)** Fluorescence microscopy to evaluate macrophage (green) phagocytosis of tumor cells (red) after different viral treatments. **(E)** Flow cytometric analysis of macrophage phagocytosis after different viral treatments. **(F)**. Tumor-associated macrophage typing after oAd-SA infection of A20 cells. Statistical significance is denoted as follows: *p < 0.05, **p < 0.01, ***p < 0.001, and **** p < 0.0001.

A subsequent study was conducted in order to confirm the results of the initial investigation, which had revealed the presence of high levels of CD47 expression in normal mouse tissues (**Supplementary Figure S1C**), as well as in mouse tumor tissues. This was achieved by obtaining relevant data from the MGI database (50), specifically 4T1, A20, and MC38, as vividly depicted in **Figure 2A**. This initial observation laid the groundwork for our subsequent inquiry into the functionality and efficacy of the SIRPα mutant secreted by tumor cells infected with the engineered oAd-SA. Employing the sophisticated technique of flow cytometry, we observed a remarkable reduction in CD47 expression on the aforementioned tumor cell lines post-infection with oAd-SA, in stark contrast to their untreated counterparts or those exposed to the control virus oAd-ON, with the 4T1 cell line exhibiting particularly pronounced effects as illustrated in **Figures 2B, C**. Further exploration through immunofluorescence and flow cytometry unveiled that infection with oAd-SA significantly bolstered the phagocytic activity of macrophages against the A20 tumor cells when juxtaposed with the control virus oAd-ON (**Figures 2D, E**). This enhancement in phagocytosis is a testament to the biological impact of the SIRPα mutant. The collective findings from these assays converge to suggest that the SIRPα mutant released by oAd-SA-infected tumor cells not only retains its functionality but also exerts a potent effect on modulating the tumor-immune cell interaction. The A20 and macrophage co-culture experiments demonstrated that oAd-SA infection resulted in an augmentation of the proportion of M1-type macrophages (**Figure 2F**).

### 3.3 SIRPα mutant augments the anticancer effects of oncolytic adenovirus *in vivo*

**Figure 3:**
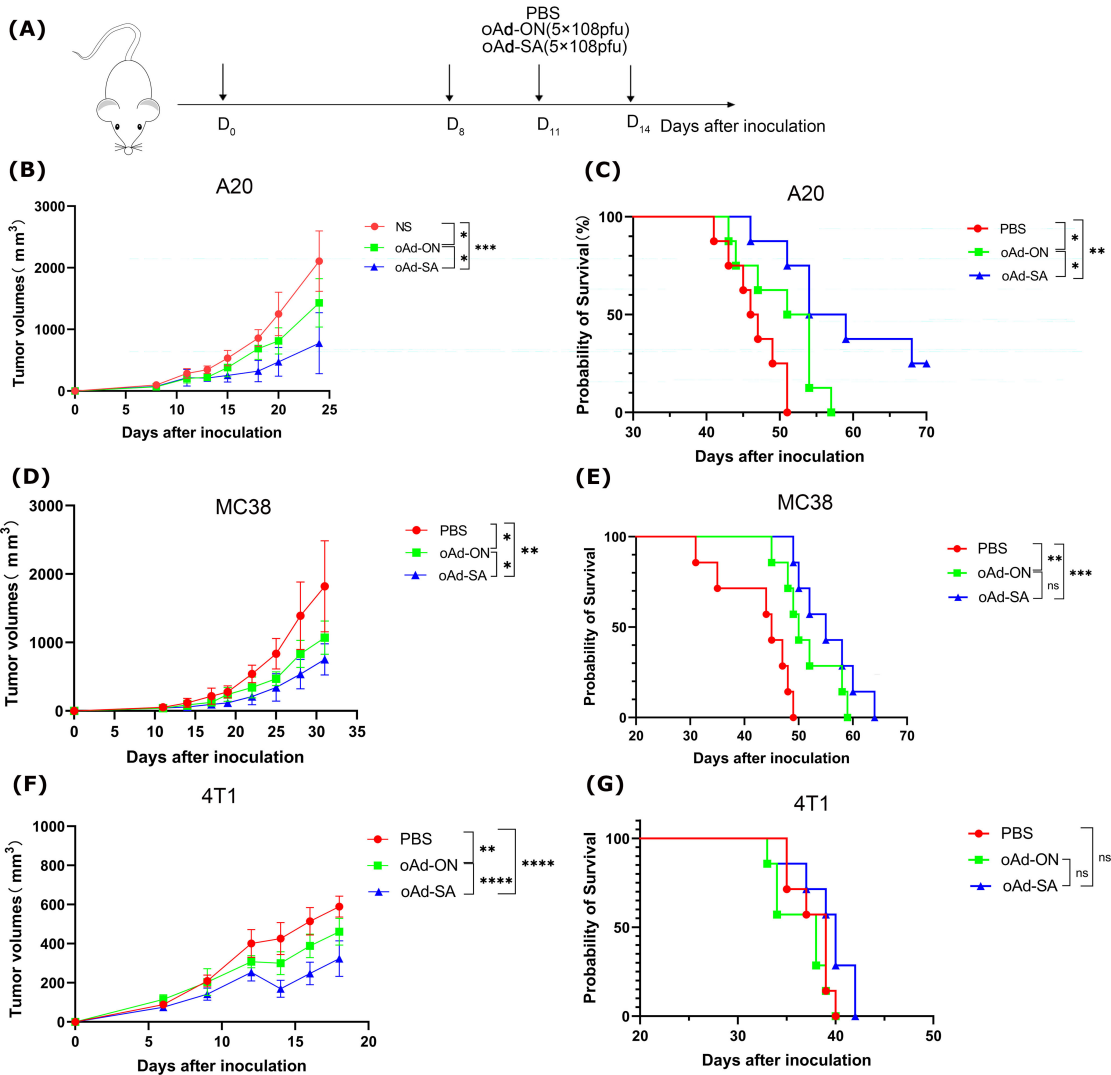
The antitumoral effects of oAd-SA *in vivo*. **(A)** Establishment of different tumor treatment models. Develop treatment models using A20, MC38, and 4T1 tumor cell lines in mice. **(B, D, G)** Tumor growth curves post-treatment in A20, MC38, and 4T1 mouse models (n=6). Analyze tumor growth in these models after treatment, and evaluate differences using the t-test. **(C, E, F)** Survival analysis post-treatment in A20 (n=8), MC38 (n=7), and 4T1 (n=7) mouse models. Statistical significance is indicated as follows: *P<0.05, **P<0.01, ***P<0.001, ****P<0.0001. "ns" for p > 0.05, means "not significant".

To meticulously assess the therapeutic potency of oAd-SA, we meticulously established subcutaneous tumor models using the A20, MC38, and 4T1 cell lines, which are representative of the malignancies under investigation. The experimental mice were subjected to a regimented treatment protocol as delineated in **Figure 3A**, ensuring a standardized approach to evaluating the intervention. In a stark contrast to the cohorts that received either PBS or the control virus oAd-ON, the intratumoral administration of oAd-SA was observed to markedly decelerate the progression of A20 subcutaneous tumors. This intervention also yielded a significant extension in the survival span of the treated animals, as evinced in **Figures 3B, C**. While all mice in the PBS and oAd-ON groups succumbed to the disease within a 57-day period post tumor cell inoculation, a notable subset of the oAd-SA treated group, specifically 3 out of 8 animals, remained alive up to 66 days post inoculation, as depicted in **Figure 2C**. Furthermore, oAd-SA demonstrated an enhanced capacity to curb the growth of MC38 and 4T1 subcutaneous tumors in comparison to the oAd-ON treatment group, as illustrated in **Figures 2D, F**. However, this therapeutic impact did not translate into a statistically significant difference in the survival outcomes for these tumor models, as indicated in **Figures 3E, G**. The aggregate data from these experiments substantiates the superior efficacy of oAd-SA in tumor inhibition when juxtaposed with the control virus oAd-ON, thereby highlighting its potential as a promising therapeutic agent in oncology. It is imperative to note that, thus far, this treatment has not exhibited any signs of tissue toxicity. Furthermore, histopathological analysis of tissue sections from mice revealed the absence of significant lesions (**Supplementary Figure S2**), thereby substantiating the remarkable safety profile of the method.

### 3.4 oAd-SA promotes the infiltration of T lymphocytes within tumor tissue and stimulates immune responses against tumor

**Figure 4:**
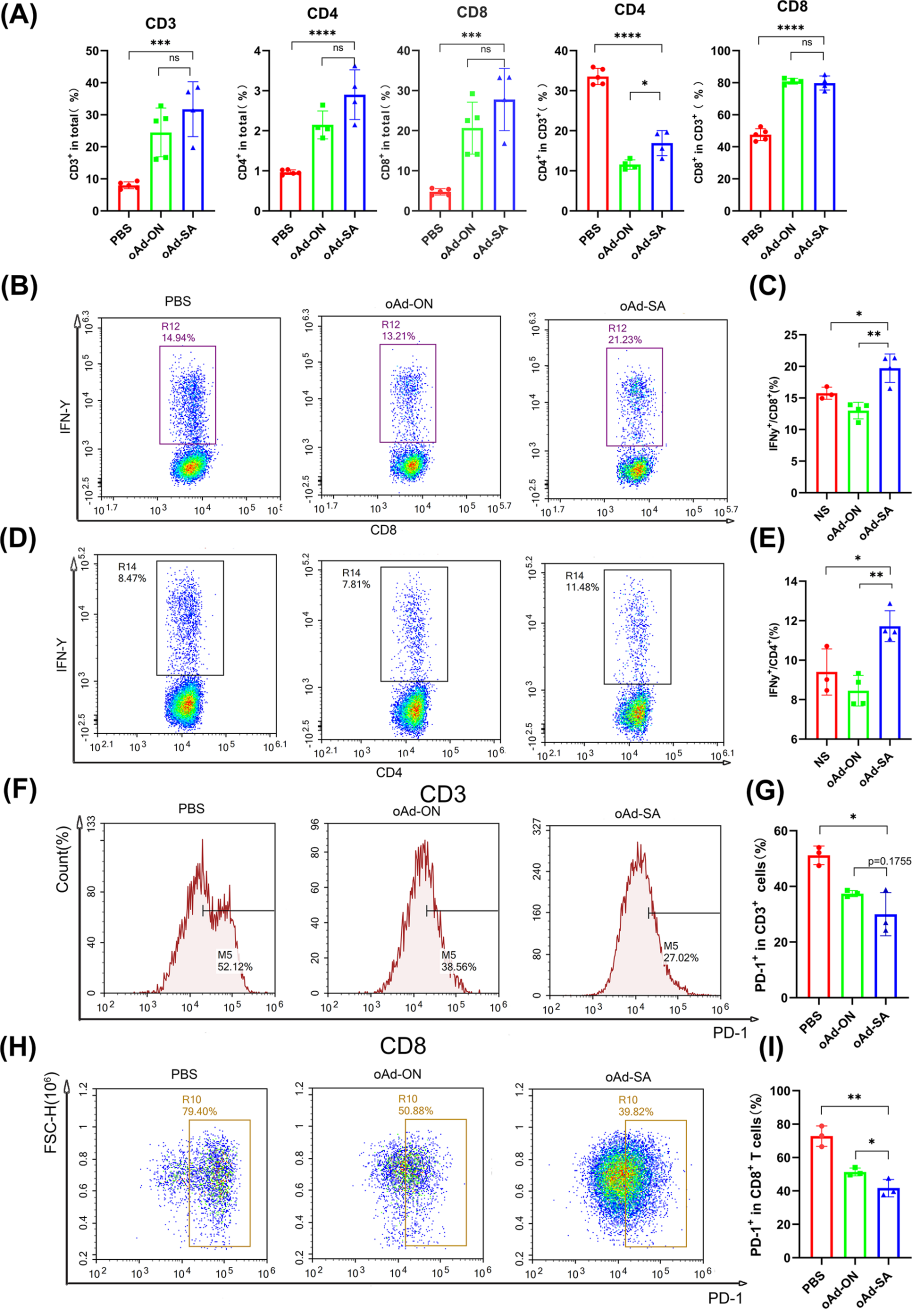
oAd-SA promotes T cells infiltration within MC38 subcutaneous tumor microenvironment and optimizes their activity and status. **(A)** Statistical analysis of CD3^+^, CD4^+^, and CD8^+^ T cell counts post-treatment in the MC38 model. **(B, D)** Flow cytometry dot plots showing IFN-γ secretion in CD8^+^ T cells and CD4^+^ T cells. **(C, E)** Statistical analysis of IFN-γ secretion in CD8^+^ and CD4^+^ T cells. **(F–I)** Proportion of PD-1^+^ cells in T cells post-viral treatment in the MC38 subcutaneous tumor model. Statistical significance is indicated as follows: *P<0.05, **P<0.01, ***P<0.001, ****P<0.0001. "ns" for p > 0.05, means "not significant".

for the purpose of investigating anticancer mechanism of oAd-SA, T cells profiles in MC38 tumor and spleen tissue was determined by flow cytometry. The results demonstrated that oAd-SA increased CD3^+^, CD4^+^ and CD8^+^ T cells percentage in all viable cell digested from MC38 tumor tissue compared with mice giving PBS. Nevertheless, oAd-SA displayed slightly higher T cells percentage than oAd-ON, no statistical significance was achieved. Moreover, it is found that oAd-SA was able to shift CD4^+^ T cells to CD8^+^ phenotype in CD3^+^ T cells. OAd-ON showed the same trend (**Figure 4A**).

T cells play an important role in overall immune response against tumor. Hence, we stimulated spleen cells with PMA and Golgi blockers for 2 h and checked IFN-γ secretion of CD4^+^ and CD8^+^ T cells from spleen. The result showed oAd-SA induced expansion IFN-γ-producing CD4^+^ and CD8^+^ T cells compared with control virus (**Figures 4B–E**), which suggests oAd-SA can activate overall immune response against tumor cells. Since antitumor effects of cytotoxic T cell could be compromised by expression of immune checkpoint molecule like PD-1, Tim-3 and CTLA-4. We further checked PD-1 expression on CD3^+^ and CD8^+^ T cells from tumor tissue. It was found that both oAd-ON and oAd-SA are capable of downregulating PD-1 expression on CD3^+^ and CD8^+^ T cells (**Figures 4F–I**). And oAd-SA can further suppress PD-1 expression on CD8^+^ T cells compare to oAd-ON (**Figures 3H, I**). These data tell oAd-SA can stimulate overall immune response against tumor and inhibit the expression of immune checkpoint on infiltrated T cells.

### 3.5 oAd-SA alters tumor-associated macrophages in tumor microenvironment and augments the phagocytosis *in vivo*

**Figure 5:**
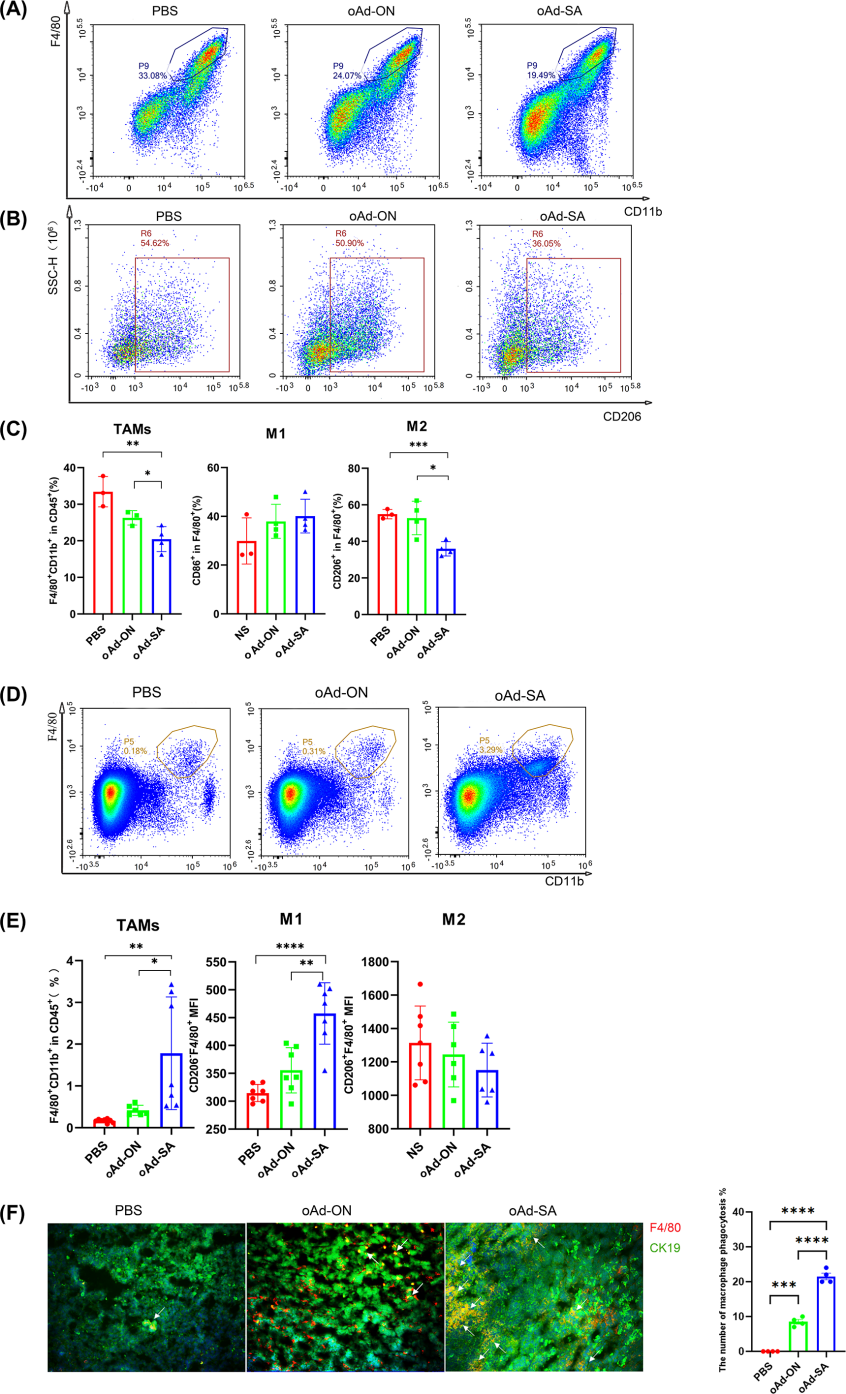
oAd-SA alters M2 phenotype TAMs into M1 phenotype within tumor tissue and promotes phagocytosis of TAMs against tumor *in vivo*. **(A, B)** Flow cytometry density plots illustrating the distribution of TAMs and M2 macrophages within the tumor microenvironment. Visualize the distribution patterns of tumor-associated macrophages (TAMs) and M2 macrophages using flow cytometry density plots. **(C)** Statistical analysis of TAMs, M1, and M2 macrophage populations post-treatment. **(D, E)** Flow cytometry density plots and statistical analysis of TAM distribution in the A20 tumor model. **(F)** Immunofluorescence analysis of macrophage phagocytosis in the 4T1 tumor model. Employ immunofluorescence to assess macrophage phagocytic activity within the tumor microenvironment of the 4T1 tumor model. Green fluorescence indicates 4T1 tumor cells, red fluorescence marks macrophages, and yellow fluorescence, as shown by overlapping signals, represents phagocytosis, as indicated by the arrows.

Given the result that oAd-SA is in position to enhance phagocytosis of macrophages against malignant cells *in vitro*, we explored the percentage and phenotypes of macrophage in tumor microenvironment with flow cytometry. The results demonstrated that oAd-SA significantly decreased the ratio of TAMs (F4/80^+^) in MC38 tumor tissue compared to mice receiving PBS and oAd-ON (**Figures 5A, C**). Most importantly, oAd-SA downregulated the percentage of M2 phenotypes (CD206^+^) of TAMs (**Figures 5B, C**). However, data from A20 tumor tissue displayed opposite result that oAd-SA increased TAMs percentage in tumor microenvironment compare with mice treated with PBS or control virus. Nevertheless, the ratio of M2 phenotype was similar among three groups. oAd-SA significantly increased the percentage of M1 phenotype which resulting in elevated M1/M2 ratio (**Figures 5D, E**). These data suggested that oAd-SA reprogrammed tumor microenvironment via altering the phenotype of macrophage. Then, we investigated if oAd-SA infection can augment phagocytosis of TAM *in vivo*. OCT frozen tissue sections of A20 tumor were stained by murine CK19 and F4/80 antibodies. Immunofluorescence result indicated that oAd-SA substantial improved phagocytotic effects of TAMs compare to oAd-ON (**Figure 5F**).

### 3.6 oAd-SA upregulates PD-L1 expression in tumor cells, yet PD-L1 antibody shows no enhancement of antitumor effects

**Figure 6:**
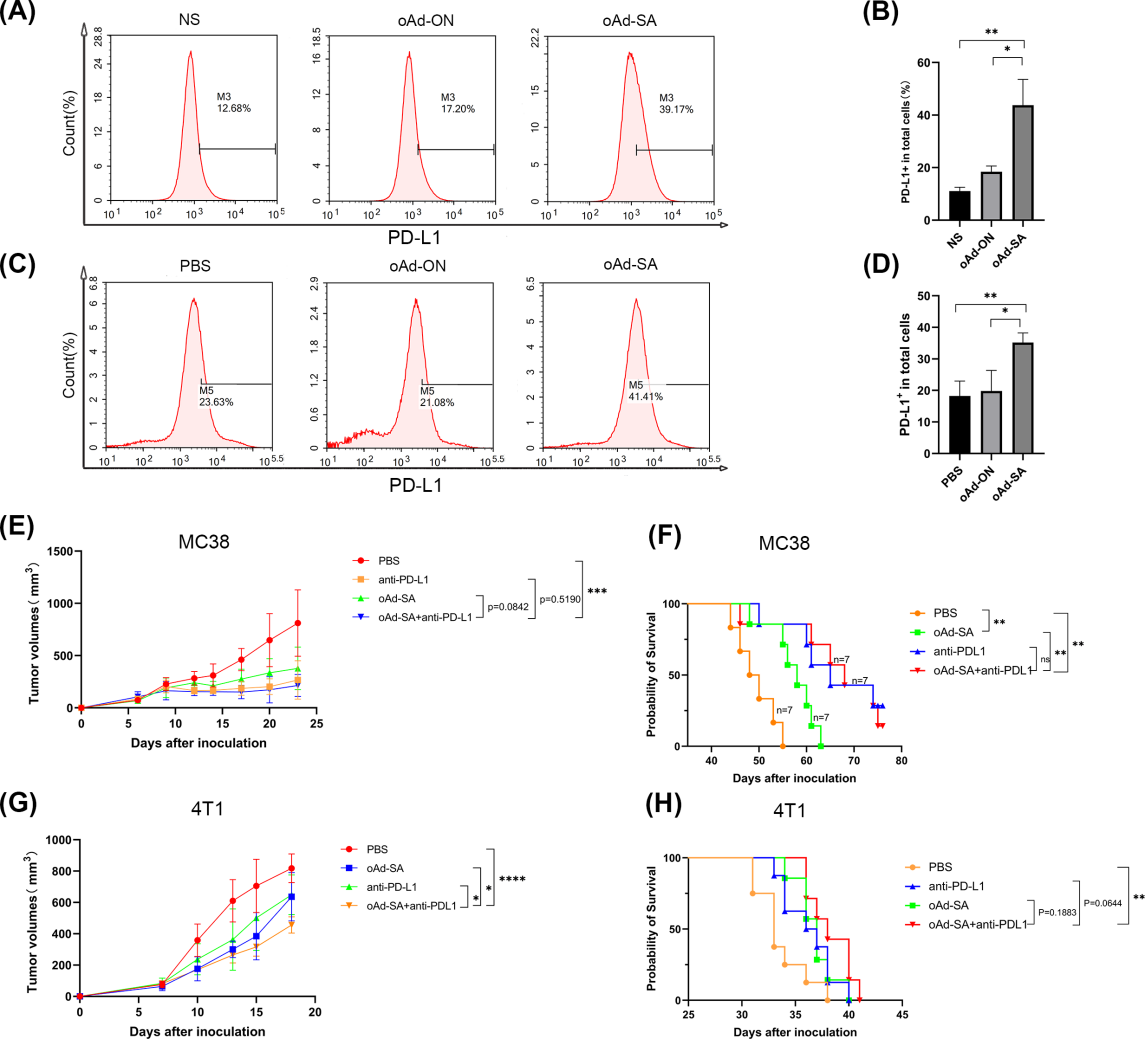
The therapeutic evaluation of oAd-SA in combination with anti-PD-L1 antibodies. **(A, B)** Analysis of PD-L1 Expression on MC38 Cells Post-Viral Infection. **(C, D)** Proportion of PD-L1^+^ Cells in the Tumor Microenvironment Post-Viral Treatment in MC38 Subcutaneous Tumor Model. **(E, F)** Tumor Growth and Survival Analysis in MC38 Model: PD-L1 antibody monotherapy significantly suppressed tumor growth and improved survival in the MC38 subcutaneous xenografts. However, adding oAd-SA to the PD-L1 antibody did not enhance these effects. **(G, H)** Tumor Growth and Survival Analysis in 4T1 Model: While the combination of oAd-SA and PD-L1 antibody led to better tumor inhibition compared to either treatment alone, it did not result in improved survival.

There are studies suggest that tumor cells would upregulate PD-L1 expression following oncolytic virus infection with the aim of escaping immune clearance. Therefore, we investigate PD-L1 level post administration of oAd-SA. Compared to animals from PBS and oAd-ON group, oAd-SA did improve PD-L1 expression in tumor microenvironment (**Figures 6A–D**). Hence, for achieving best therapeutic effect, we treated mice with oAd-SA in combination with PD-L1 antibody. Yet, the results were rather disappointing. In MC38 subcutaneous xenografts, animals were sensitive to PD-L1 antibody monotherapy. Mice administrated with combination treatment of oAd-SA and PD-L1 antibody displayed no gain on both tumor suppression and survival compared to ones receiving PD-L1 antibody alone (**Figures 6E, F**). In 4T1 model, despite combination of two agents showed superior tumor inhibition capability compared with oAd-SA or PD-L1 monotherapy, no improvement was achieved on animal survival. (**Figures 6G, H**).

## 4 Discussion

Tumor-associated macrophages (TAMs) constitute a significant proportion of the immune cell infiltrate within neoplastic tissues, playing a dual role in the tumor microenvironment (51). These cells can be dichotomously classified based on their functional phenotypes: the M1-like phenotype, which is characterized by its antitumorigenic properties, is instrumental in antigen presentation and promoting a Th1-type immune response, while the M2-like phenotype is associated with pro-tumorigenic functions, including the facilitation of tumor growth, suppression of T cell activity, and a correlation with poor therapeutic outcomes (52–54).Recent studies have illuminated that the dual approach of blocking the CD47/SIRPα interaction and employing oncolytic viruses can profoundly restructure the M1/M2 macrophage balance, driving a phenotypic shift from the immunosuppressive M2 state to the pro-inflammatory M1 state (55–57). Moreover, the disruption of the CD47/SIRPα interaction has been shown to directly enhance the antitumor response by stimulating the phagocytic activity of TAMs (57).Consistent with these findings, our data reveal that the oAd-SA treated group exhibited the highest M1/M2 ratio among all experimental groups, underscoring the therapeutic potential of this approach. Additionally, the SIRPα-Fc fusion protein, as produced by oAd-SA, has been demonstrated to be functionally active, augmenting the phagocytic capacity of macrophages in both *in vitro* assays and *in vivo* models.

Exploiting the immune system to clear tumor cells has demonstrated drastic curative efficacy in various malignancies treatment. The therapeutic efficacy of immunotherapy universally relies on combination of innate and adaptive antitumor responses (58, 59). Blockade of CD47 strengthens antibody-dependent cellular phagocytosis (ADCP), which result in the release of cytotoxin and direct engulfment. In turn, ADCP of macrophages and dendritic cells (DCs) triggers tumor-specific antigen processing and presentation, priming effector T cells differentiation and expansion. For example, anti-CD47 antibody-regulated phagocytosis of tumor cells by macrophages primes the proliferation of CD8^+^ T cells both *in vitro* and *in vivo* and lead to reduction of FoxP3^+^ regulatory T cells (Tregs) *in vitro (* 60). Moreover, anti-CD47 treatment reinvigorate effector T cells in head and neck squamous cell carcinoma mouse model and alters tumor microenvironment via reducing the infiltration of Tregs and myeloid-derived suppressor cells (MDSCs) and decrease the suppressive function of MDSCs (61). CD47 blockade triggers the cross-priming capability of DCs, thus initiates T cell-mediated inhibition of immunogenic tumors (62). These studies demonstrated that anti-CD47 antibodies can stimulate antitumor T cells response and modulating immunosuppressive microenvironment to protect animals from tumor challenge. Nevertheless, inhibition of CD47/SIRPα interaction monotherapy is insufficient to control tumor progression in some cancers which will require combination treatment to achieve synergistic effects (20, 63–66).

Meanwhile, oncolytic adenovirus is known to remodel tumor microenvironment and turn the poor T lymphocytes infiltrated “cold tumor” into “hot tumor” characterized by larger proportion of T cells infiltration (9, 67). Therefore, oAd-SA treatment may elicit a stronger tumor-targeting immune response as compared to anti-CD47/SIRPα antibodies alone or control virus. In the present study, it is found that oAd-SA slightly upregulated the percentage of T lymphocytes including CD3^+^, CD4^+^ and CD8^+^ T cells within tumor tissue as compared to oAd-ON, but no statistical significance was achieved between these two groups. Nevertheless, oAd-SA increased the anti-tumor activity of T cells as compared to oAd-ON, this was evidenced by upregulation of the ratio of IFN-γ-secreting CD4^+^, CD8^+^ T cells in spleen of mice treated with oAd-SA as compared to oAd-ON receiving animals.

In addition, the functionality of CD8^+^ T cells is important in protective immunity against tumors. In OVs treatment, CD8^+^ T cells are constantly exposed to tumor antigens and inflammatory signals which results in T cells exhaustion characterized by expression of multiple inhibitory molecules including PD-1, Tim3 and LAG3 (68, 69). Also, an oncolytic vaccinia virus triggers PD-L1 expression on both immune and cancer cells within tumor tissue (70). In the present study, oAd-ON therapy significantly reduced PD-1 expression on CD3^+^ and CD8^+^ T cells in tumor microenvironment. The inhibitory effect was further augmented by treatment of oAd-SA, especially on PD-1 expression on CD8^+^ T cells. Moreover, injection of oAd-SA drastically upregulated PD-L1 expression on cells collected from tumor tissue as compared to oAd-ON and PBS, which is consistent with study we mentioned before (70).

There has been a growing interest in combining anti-PD-L1 antibodies with OVs or CD47/SIRPα blockades to improve therapeutic efficacy. Studies have demonstrated that dual inhibition of CD47 and PD-L1 induced complete tumor progression in murine models (71, 72). Likewise, combination treatment of different OVs and anti-PD-L1 antibodies resulted in synergistic and durable antitumor effects in both preclinical and clinical studies (73–76). In this study, given the result that oAd-SA largely upregulated PD-L1 expression on cells from tumor tissue, the therapeutic efficacy of oAd-SA in combination with anti-PD-L1 antibody was investigated. Combination treatment displayed superior tumor inhibition in 4T1 subcutaneous tumor model as compared to monotherapy.

In conclusion, the SIRPα mutant engineered by oAd-SA has demonstrated remarkable efficacy, achieving a substantial reduction in CD47 expression across a variety of mouse tumor cell lines. This downregulation of CD47 expression serves as a pivotal mechanism that significantly amplifies the phagocytic activity of macrophages against tumor cells, thereby reinforcing the immune system’s innate capacity to combat malignancy. The intratumorally administration of oAd-SA has been shown to effectively restructure the tumor microenvironment through a multifaceted approach. It enhances the infiltration of CD3^+^, CD4^+^, and CD8^+^ T cells, invigorating the immune response at the site of the tumor. Furthermore, oAd-SA treatment strategically targets and reduces the expression of PD-1 on CD8^+^ T cells, rejuvenating their antitumor functionality and countering the exhaustion often observed in T cells within the tumor microenvironment. Additionally, oAd-SA facilitates a critical shift in the polarization of tumor-associated macrophages (TAMs), transitioning the pro-tumorigenic M2 phenotype to the antitumor M1 phenotype. This transformation is instrumental in reestablishing a balanced and effective immune response against the neoplastic cells. Importantly, treatment with oAd-SA has been correlated with a significant delay in tumor progression and an extension of survival in tumor-bearing animals, outperforming the effects of the control virus. These findings underscore the potential of oAd-SA as a promising therapeutic agent in the realm of oncology.

## Data Availability

The original contributions presented in the study are included in the article/**Supplementary Material**. Further inquiries can be directed to the corresponding author.

## Supplementary material

Supplementary Figure 1
**(A)** Statistical analysis of differences in CD3^+^, CD4^+^, and CD8^+^ T cell counts after treatment was performed using the t-test method. *P < 0.05, **P < 0.01, ***P < 0.001, ****P < 0.0001. **(B)** Green fluorescence indicates 4T1 tumor cells, red fluorescence marks macrophages, and the overlapping yellow fluorescence represents phagocytosis, as indicated by the arrows. **(C)** Expression of CD47 in normal tissues of mice from the MGI database, taken as TPM values. **(D)** Therapeutic effect of different doses of oAd-SA on mouse tumors (Low = 1*10^8^ pfu; Medium = 5*10^8^ pfu; High = 2.5*10^9^ pfu).

Supplementary Figure 2 Safety evaluation of oAd-SA virus (bar = 50 µm).
