# Supplementary figures and images for: Expression of SIRPα-Fc by oncolytic virus enhances antitumor efficacy through tumor microenvironment reprogramming
Source: Front Immunol. 2025 Feb 25;16:1513555. doi: 10.3389/fimmu.2025.1513555 (PMC11893986; doi:10.3389/fimmu.2025.1513555)

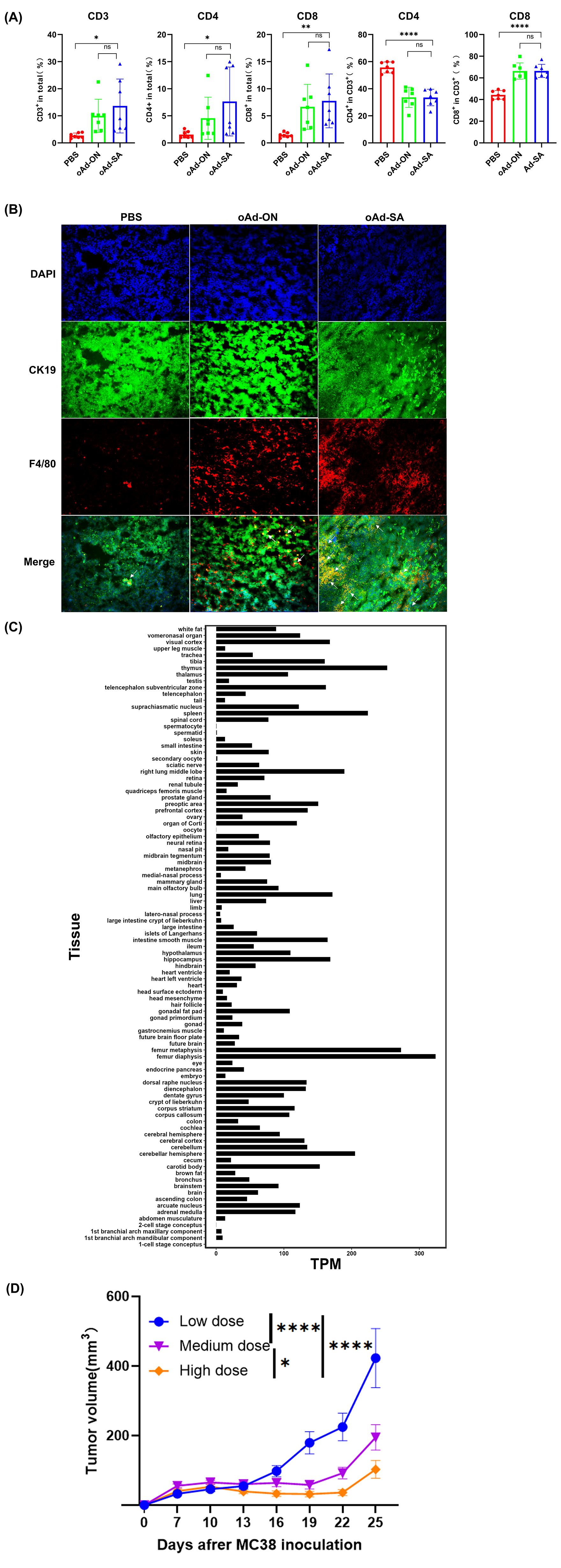

Supplement: Supplementary Figure 1 — (A) Statistical analysis of differences in CD3+, CD4+, and CD8+ T cell counts after treatment was performed using the t-test method. *P < 0.05, **P < 0.01, ***P < 0.001, ****P < 0.0001. (B) Green fluorescence indicates 4T1 tumor cells, red fluorescence marks macrophages, and the overlapping yellow fluorescence represents phagocytosis, as indicated by the arrows. (C) Expression of CD47 in normal tissues of mice from the MGI database, taken as TPM values. (D) Therapeutic effect of different doses of oAd-SA on mouse tumors (Low = 1*108 pfu; Medium = 5*108 pfu; High = 2.5*109 pfu). [file Image1.tif]

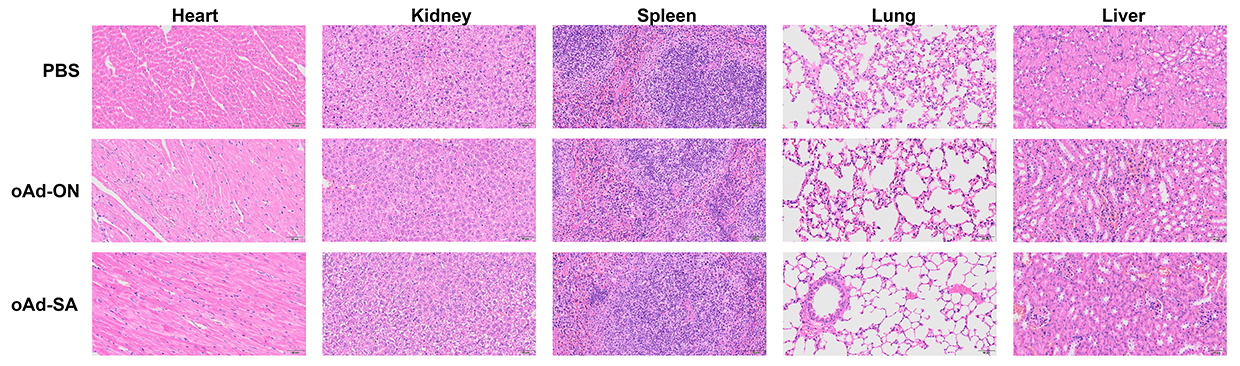

Supplement: Supplementary Figure 2 — Safety evaluation of oAd-SA virus (bar = 50 µm). [file Image2.tif]
